# Importance of the Motivational Climate in Goal, Enjoyment, and the Causes of Success in Handball Players

**DOI:** 10.3389/fpsyg.2017.02081

**Published:** 2017-12-01

**Authors:** Antonio Granero-Gallegos, Manuel Gómez-López, Nuria Rodríguez-Suárez, J. Arturo Abraldes, Marianna Alesi, Antonino Bianco

**Affiliations:** ^1^Department of Education, University of Almería, Almería, Spain; ^2^Department of Physical Activity and Sport, University of Murcia, Murcia, Spain; ^3^Department of Science of Physical Activity and Sport, Catholic University San Antonio of Murcia, Murcia, Spain; ^4^Department of Psychological, Pedagogical and Education Sciences, University of Palermo, Palermo, Italy

**Keywords:** perceived motivational climate, goal orientation, sport satisfaction, emotions, handball

## Abstract

The purpose of the study is to examine the effects of the motivational climate created by the coach and perceived by a group of young handball players on their goal orientations, their beliefs regarding reasons for success and their self-satisfaction. The study participants were 159 young handball players. Players were administered a battery composed of tests to measure the above-mentioned motivational constructs. Results showed that a perceived mastery-oriented motivational climate was positively related to a task-centered goal orientation, enjoyment, and a belief that success may be achieved through effort. In contrast, a perceived performance-orientated training climate was linked to an ego-centered goal orientation, boredom, or lack of enthusiasm, and a belief that the routes of success in this sport are the abilities and the use of deception techniques. On the whole, this study underlines the educational role of the coach in young handball players. Specifically, the coach’s socializing role influences both handball player’s commitment and positive motivational profile as well as sport performance.

## Introduction

Coaches, parents, peers, and media all play a key role in sport motivation by contributing to influence young or adult athlete performance. Studies in educational psychology have demonstrated how the achievement in social competitive contexts, such as school or sport domains, depends on the reciprocal enhancement of abilities and emotional–motivational attributes (Pepi et al., 2006; Alesi et al., 2015).

A current line of research examines the important role played by the coach and its impact on the motivational climate in the team following the framework of achievement goal theory (Duda, 2001; Duda and Balaguer, 2007; Torregrosa et al., 2008; Granero-Gallegos et al., 2012; Gómez-López et al., 2013, 2014; Nicholls et al., 2016). In particular, coaches interact for many hours with their players both in training sessions and competition periods (Ruiz Sánchez et al., 2017). This determines the coach is an essential educational figure for player quality during a sport’s career (Gagné et al., 2003). The Social Cognitive Theory of achievement goal perspectives proposed that athletes are motivated by the need to show their competence and avoid displaying their incompetence (Cervelló and Santos-Rosa, 2001; Cecchini et al., 2004). The use of these criteria to define success or failure depends as much on athletes’ personal characteristics (dispositional orientation) as on their social environment (motivational climate). Specifically, the achievement goal theory (Nicholls, 1989; Ames, 1992) underlies the individuals’ disposition to demonstrate the own ability and competence when participating in the achievement context. Two different achievement goals, namely task and ego involvement (Nicholls, 1989) drive their ability perception. As a consequence, two goal orientations known as ego- or performance-orientated and as task- or mastery-orientated can be differentiated. A task goal orientation determines adaptive motivational, affective, and behavioral patterns that are more positive than an ego goal orientation (Duda, 2001). Thus, task-oriented athletes judge their competence level by self-comparison and view sport as reinforcing their cooperation capacity and interest in learning. In contrast, ego-oriented athletes judge their competence by comparing themselves with others and consider sports’ practice as a way to gain recognition and social status (Nicholls, 1989; Cervelló et al., 1999; Duda, 2001; Ferreira et al., 2016). These athletes attribute failure to a lack of ability, skill, and effort (Treasure and Roberts, 2001).

Studies have also shown that goal orientations are good predictors of beliefs about the reasons for success in sport (Treasure and Roberts, 1994; Cervelló et al., 1999) and of enjoyment or self-satisfaction with sports’ practice (Duda et al., 1995; Cervelló et al., 1999; Cecchini et al., 2004; Granero-Gallegos et al., 2014). This means that task-orientated athletes are more likely to attribute their success to their efforts. Moreover, they tend to consider failure as a learning experience to improve their skills and performances, rather than a negative or frustrating experience. This perception will have the consequence of enjoyment and satisfaction along with a greater commitment to the given sport. On the other hand, athletes with an ego-orientated represent the success as achievable through ability and deception. They see the failure as an indicator of limited abilities. In this condition, they will quickly become bored or disillusioned with the given sport’s activity and show a lower level of personal satisfaction. In consequence, ego-orientated athletes will be more likely to drop-out the sport, especially if sport challenges and results are perceived as an indicator of their limited abilities (Duda et al., 1995; Cervelló and Santos-Rosa, 2001; Castillo et al., 2002; Walling et al., 2002; Cecchini et al., 2004). Moreover, the nature of the sport is hypothesized to influence the adoption of goal orientations. Individual sports are more informative about personal performance; this lead athletes to adopt ego-oriented goals aimed at ensuring positive judgments of their skills, with comparative and normative evaluations of personal performance, as well as to have the need to show themselves and others their personal success as an indicator of personal high ability. Furthermore, this level of ego-orientation was found to be higher in the competition context than in the training one because of the psychological pressure (Van de Pol and Kavussanu, 2012).

Individual goal orientations may be influenced or modulated by the motivational climate perceived by the subject (Nicholls, 1989; Ames, 1992; Jaakkola et al., 2016). Schneider and Reichers (1983) carry out a review of studies concerning the climate etiology. The authors focus on conceptual and methodological progress in this research topic and argue that individuals attribute meanings to events through interactionism processes with the context over the time. According to Ames (1992), the motivational climate can be described as a set of implicit and/or explicit environmental signals through which the keys to success or failure are defined. So, in the field of sports the individual-level climate perceptions are strongly influenced by the individual goal orientations as well as the educational figure of the coach (Nicholls, 1989; Ames, 1992; Seifriz et al., 1992; Escartí et al., 1999; Newton et al., 2000; Cervelló and Santos-Rosa, 2001; Curran et al., 2015). A performance climate will be created when the most important coach’s goals are that the team wins, the demonstration of skills and effort, the punishment of errors promoting inter-individual competition, and rivalry between team members. This climate induces anxiety regarding performance and reduced satisfaction with the sport’s environment. In contrast, when the coach promotes learning through errors, personal progress, skill development, and cooperation between team members, the climate will be one of mastery, inducing the psychological well-being of the athlete (Balaguer et al., 1999, 2002; Newton et al., 2000; Pensgaard and Roberts, 2000; Duda, 2001; Atkins et al., 2015). A mastery-oriented motivational climate is positively related to task-orientated goals, enjoyment, satisfaction, interest, self-motivation, and commitment rather than the negative affective behavior and feeling of pressure associated with a performance-orientated motivational climate. Accordingly, the ego or mastery perspective of an athlete at a given time will be determined by the perceived motivational climate, goal orientations, and the environment characteristics (Nicholls, 1989).

The present study examines the effects of the motivational climate created by the coach and perceived by a group of young handball players on their goal orientations, their beliefs regarding reasons for success, and their self-satisfaction. It was hypothesized that a perceived mastery-oriented climate was positively related to a task-centered goal orientation, personal enjoyment, and the belief that success is achieved through effort while a perceived performance-orientated climate was related to an ego-based orientation, boredom, and the belief that ability and deception are means to success.

## Materials and Methods

### Sample

The subjects enrolled were 159 handball players. Participants were youth to senior category (age: *M* = 18.23; *SD* = 4.02; age range: 12–28 years old) whose clubs participated in national Youth, Junior, and Mixed championships: 90 were male (*M* = 18.81; *SD* = 4.32) and 69 female (*M* = 17.60; *SD* = 3.65). All participants were trained three times a week for 60–90 min per session, in addition to the competition at the weekend. The study was approved by the Ethics Committee of the University of Murcia.

### Instruments

#### Perceived Motivational Climate in Sport Questionnaire (PMCSQ-2)

To assess perceived motivational climate, we used the Spanish version (Balaguer et al., 1997) of this questionnaire (Newton and Duda, 1993). This scale contains 29 items divided into two dimensions’ performance and mastery assessing ego-involvement climate (14 items) and task-involvement climate (15 items), respectively. The stem for each question was “In my handball team ....” An example of a task-involving dimension item is “The coach encourages players to help each other.” An example of an item reflecting an ego-involving dimension is “Only the top players ‘get noticed’ by the coach.” The subjects’ responses were indicated on a five-point Likert-type scale (1 = strongly disagree; 5 = strongly agree). The internal consistency of this questionnaire emerged as satisfactory both for the subscales mastery (α = 0.87) and performance (α = 0.87).

#### Task and Ego Orientation in Sport Questionnaire (TEOSQ) (Duda, 1989; Balaguer et al., 1996)

We used the Spanish version with 13 items designed to assess a person’s approach to sport as more ego-orientated (six items) or task-orientated (seven items). The TEOSQ requested the subjects to think about when they felt most successful in handball and to respond to 13 items designed to assess task-involved criteria (e.g., “I feel most successful in handball when something I learn makes me want to practice more”) and ego-involved criteria (e.g., “I feel most successful in handball when others mess up, and I don’t”) for defining success. The subjects’ responses were indicated on a five-point Likert type scale (1 = strongly disagree; 5 = strongly agree). Internal consistency was satisfactory: ego-orientated, α = 0.87; task-orientated, α = 0.73.

#### Sport Satisfaction Instrument (SSI) (Duda and Nicholls, 1992; Balaguer et al., 1997; Castillo et al., 2002)

The original questionnaire has eight items divided into two scales that assess satisfaction/enjoyment (five items) and boredom (three items) in sport. Examples of items included, satisfaction/enjoyment: “I have fun doing sport,” boredom: “In sport, I often daydream instead of thinking about what I really do.” The subjects’ responses were indicated on a five-point Likert-type scale (1 = strongly disagree; 5 = strongly agree). The internal consistency indices recorded were: satisfaction/enjoyment, α = 0.80; boredom, α = 0.60. Although this latter factor showed a reliability or alpha below the recommended value of 0.70 (Nunnally, 1978; Peterson, 1994), its small number of comprising items (three) determines that this internal validity is marginally acceptable (Nunnally and Bernstein, 1994; Hair et al., 1998).

#### Beliefs about the Causes of Success in Sport Questionnaire (BACSSQ) (Duda and Nicholls, 1992; Balaguer et al., 1997; Castillo et al., 2002)

The original questionnaire contains 18 items assessing beliefs about whether effort (nine items, effort exerted in the performance of the task), ability (four items, factors related with the ability possession), and the use of deception techniques (five items, making use of deceptive behavior) lead to success in sport. In the instructions, the participants were asked: “What do you think people should do in order to be successful in the sport they practice more often?” The general stem to each item was “Kids who play handball succeed if they ... .” Examples of items included: effort: “... work really hard”; ability “... are born natural athletes”; and deception techniques: “... pretend to like the coach.” The subjects’ responses were indicated on a five-point Likert-type scale (1 = strongly disagree; 5 = strongly agree). Internal consistency was satisfactory: effort, α = 0.87, ability, α = 0.80; deception techniques, α = 0.89.

### Procedure

Approval for the study was obtained from the Handball Federations and participating clubs, according with the rules of the University of Murcia. These institutions were contacted by email in order to explain both the study aims and procedures and, additionally, to provide them an example of the questionnaire. The questionnaire was administered the day before a national competition during the training sessions of the teams involved.

All participants were informed about the study objectives, the voluntary nature of their participation, the confidentiality of their responses, and of data treatment. The signing of the consent form was necessary to take part in this research. It was also mentioned that there were no right or wrong answers and they should reply as honestly as possible to each question. The questionnaire requires about 30 min in total to complete. The data collection was performed following all ethical procedures.

## Results

### Data Analysis

The software package SPSS v. 17.0 was used for analysis of the items, homogeneity, correlation among subscales (Pearson coefficient), internal consistency of each subscale (Cronbach’s alpha), multivariate analysis (MANOVA 2 × 2), and canonical correlations. Confirmatory factor analysis (CFA) was performed using LISREL 8.80 (Jöreskog and Sörbom, 2003) to assess the factorial structure of each questionnaire. It was carried out as an analysis of multivariate normality of each of the scales. The normality test was performed based on the PRELIS relative multivariate kurtosis (RMK) of the LISREL program 8.80. The results of the test at each scale showed that the normality multivariate cannot be accepted, which implies the use of the robust estimators. For this reason, the weighted least squares (WLS) estimation method was used for ordinal variables of the LISREL 8.80 program. As input for data analysis, a polychoric correlation asymptotic covariance matrix was constructed. The assumption was made of the presence of latent variables according to the original instruments. The instrument models were assessed by calculating indices of relative and absolute fit. Among the absolute indices, *p*-value was used, associated with the chi-square test (χ^2^); chi-squared divided by degrees of freedom (*df*) (χ^2^/*df*); the ratio <2 would be considered as indicators of a very good model fit (Tabachnick and Fidell, 2007), while values <5 are considered acceptable (Hu and Bentler, 1999); we also calculated the *Goodness Fit Index* (GFI), which indicates the relative amount of variance and covariance reproduced by the specific model, compared to a saturated model, whose value must be equal to or >0.90 to be considered a minimally acceptable model fit, although authors such as Hooper et al. (2008) consider values ≥0.95 make a better fit. Among relatives indices were used: *Normalized Fit Index* (NFI), *Non-normative Fit Index* (NNFI), and *Comparative Fit Index* (CFI); it is considered that values ≥0.95 mean a good fit. Also, the *Root Mean Square Error of Approximation* (RMSEA) was used as incremental índice and values ≤0.06 indicate a good fit (Hu and Bentler, 1999). Significance for these indices was set at a *t*-value >1.96 (*p* < 0.05).

The CFA revealed an adequate goodness on different instruments used in this research. *PMCSQ-2*: χ^2^ = 683.80, *p* < 0.001, χ^2^/gl = 1.69, GFI = 0.98, NFI = 0.99, NNFI = 0.98, CFI = 0.99, RMSEA = 0.04; *TEOSQ*: χ^2^ = 78.27, *p* < 0.001, χ^2^/df = 1.99, GFI = 0.98, NFI = 0.97, NNFI = 0.97, CFI = 0.98, RMSEA = 0.04; *SSI*: χ^2^ = 44.99, *p* = 0.001, χ^2^/df = 2.37, GFI = 0.98, NFI = 0.96, NNFI = 0.97, CFI = 0.97, RMSEA = 0.06; *BACSSQ*: χ^2^ = 154.11, *p* > 0.001; χ^2^/df = 2.08, GFI = 0.95, NFI = 0.95, NNFI = 0.96, CFI = 0.96, RMSEA = 0.06.

### Descriptive Statistics and Group Comparisons

The data provided in **Table 1** indicate higher means recorded in the study participants for mastery (*M* = 4.07) than performance perceived motivational climates and higher means for task than ego goal orientations. The SSI also revealed much higher mean scores for enjoyment than boredom. Lastly, among the reasons for success, effort was the most highly rated followed by ability, and, lastly, the use of deception techniques.

**Table 1:** Mean, standard deviation, skewness, kurtosis, and Cronbach’s alpha coefficients recorded for the subscales examined.

Subscale	*M*	*SD*	Skewness	Kurtosis	α
**Motivational climate**					
Mastery	4.07	0.53	-0.93	2.15	0.87
Performance	2.90	0.69	0.12	-0.24	0.87
**Goal orientations**					
Ego	3.06	1.15	-0.05	-0.96	0.94
Task	4.23	0.59	-0.46	-0.30	0.80
**Satisfaction**					
Enjoyment	4.26	0.61	-0.52	-0.38	0.80
Boredom	2.51	0.92	0.73	0.24	0.60
**Reasons for success**					
Effort	4.08	0.68	-1.17	2.10	0.87
Ability	3.23	0.94	-0.17	-0.53	0.80
Deception techniques	2.17	1.17	0.71	-0.60	0.89

*M, mean; *SD*, standard deviation; α, Cronbach’s alpha.*

A MANOVA (2 × 2) was performed to examine how the goal orientation, satisfaction, and perceptions of beliefs about player’s success are based on the motivational climate perceived by athlete (high and low mastery, high and low performance). For that, the young handball players were classified into four groups according to whether their scores in the subscales mastery (median = 4.1) and performance (median = 2.8) were higher or lower than the median as follows: high-mastery (*n* = 46 men, *n* = 37 women), low-mastery (*n* = 44 men, *n* = 32 women), high-performance (*n* = 51 men, *n* = 39 women), and low-performance (*n* = 39 men, *n* = 30 women). A 2 × 2 MANOVA was then performed using two levels each of mastery motivational climate (high–low) and performance motivational climate (high–low) as independent variables, and the subscales of goal orientations, satisfaction, and reasons for success as the dependent variables.

This test revealed a non-significant multivariate effect of the interaction mastery × performance on the goal orientation subscales satisfaction, and reasons for success [Wilks’ lambda: 0.96, *F*(7,101) = 0.56, *p* = 0.790]. However, a main significant multivariate effect was observed for a perceived mastery motivational climate [Wilks’ lambda = 0.82, *F*(7,101) = 3.16; *p* = 0.005] and a perceived performance motivational climate [Wilks’ lambda = 0.85; *F*(7,101) = 2.48, *p* = 0.022]. Subsequent univariate analyses indicated that athletes who perceived the training climate as more mastery-oriented showed more task-centered goal orientations (*F* = 9.76, *p* = 0.002), more enjoyment (*F* = 16.63, *p* = 0.001), and put more effort into the training sessions (*F* = 7.94, *p* = 0.006) than participants who perceived the training climate as less mastery-oriented. Likewise, it was observed that individuals who perceived a training climate that was more performance-orientated scored higher in ego-centered goal orientations (*F* = 4.57, *p* = 0.035), boredom (*F* = 8.36, *p* = 0.005), and deception techniques (*F* = 8.46, *p* = 0.004) (**Table 2**).

**Table 2:** Univariate effect of perceived motivational climate on goal orientation, satisfaction, and reasons for success according to the level of perceived mastery and performance motivation climate.

	Mastery motivation climate					Performance motivation climate				
	High (*n* = 83) (*M*)	Low (*n* = 76) (*M*)	*F*	*p*	Partial η^2^	High (*n* = 90) (*M*)	Low (*n* = 69) (*M*)	*F*	*p*	Partial η^2^
**Goal orientations**								
Ego	2.60	2.72	0.32	0.574	0.00	2.88	2.44	4.57	0.035	0.06
Task	4.12	3.83	9.76	0.002	0.16	4.02	3.93	1.02	0.314	0.01
**Satisfaction**								
Enjoyment	4.47	4.03	16.63	0.000	0.26	4.29	4.22	0.47	0.494	0.00
Boredom	2.42	2.48	0.13	0.717	0.00	2.69	2.22	8.36	0.005	0.12
**Reasons for success**								
Effort	4.31	3.97	7.94	0.006	0.14	4.07	4.21	1.32	0.254	0.00
Ability	3.23	3.22	0.01	0.973	0.00	3.35	3.10	2.21	0.140	0.01
Deception techniques	1.90	2.07	0.67	0.414	0.02	2.28	1.68	8.46	0.004	0.11

*Differences between means significant at *p* = 0.05; *n* = sample; *M*, mean; η^*2*^, eta square.*

### Canonical Correlations

Multivariate relationships between perceived motivational climate and goal orientation, satisfaction, and reasons for success were examined by canonical correlation analysis. Two significant functions were observed. In the first function, Wilks’ lambda = 0.38; *F* = 1.61, *p* < 0.001, and rc1 = 0.78. In the second function, Wilks’ lambda = 0.36; *F* = 1.74, *p* < 0.001, and rc2 = 0.80. The high canonical correlation detected between the two functions indicates that the discriminant variables examined were able to differentiate between the high/low mastery/performance groups. In addition, according to the level of significance detected within the compared groups, the null hypothesis for the two discriminant variables was rejected. The variance explained by the linear combination of the predictor and criterion variables was 61% in the first function, and 64% in the second function. The data in **Table 3** indicate that in the first function, the mastery-oriented motivational climate showed high, positive loadings, whereas the performance-oriented climate had low, negative canonical loadings. Besides, a perceived mastery-orientated motivational climate in the training sessions was positively related to a task goal orientation, enjoyment, and effort. In the second function, high, positive canonical loadings were obtained for the performance-oriented motivational climate and low, negative loadings for the mastery climate. A perceived performance climate was mainly associated with ego-centered goal orientations, being bored in training sessions, and the use of deception techniques. Loadings higher than 0.30 were considered significant (Tabachnick and Fidell, 2007).

**Table 3:** Canonical loadings recorded between perceived motivational climate and goal orientation, satisfaction, or reasons for success.

Variable	Function 1	Function 2
**Criterion variables**		
Motivational climate		
Mastery	0.96	-0.02
Performance	-0.14	0.95
**Predictive variables**		
**Goal orientations**		
Ego	-0.34	0.36
Task	0.35	0.08
**Satisfaction**		
Enjoyment	0.42	0.06
Boredom	-0.30	0.41
**Reasons for success**		
Effort	0.30	-0.37
Ability	0.11	0.11
Deception techniques	-0.14	0.31

## Discussion

According to our descriptive analysis, the handball players examined here perceived their training climate as more orientated toward mastery than toward performance. This finding is consistent with reports by other authors (Moreno et al., 2007; Torregrosa et al., 2008).

The present canonical correlation analysis positively related a perception of a mastery-orientated training climate with a task-centered goal orientation, enjoyment, and effort, and related a perceived performance-orientated training climate with an ego-centered goal orientation, boredom, and the use of deception techniques. Accordingly, our results suggest that although the population examined featured both goal orientation perspectives, most of the handball players perceived a mastery-orientated motivational climate and a task goal orientation, reported they enjoyed their sport and felt that effort was the means to success. These results are similar to those detected by Holgado et al. (2010) for professional athletes, who described that participants developing a task-centered goal orientation found satisfaction in their sport and perceived a motivational climate that rewarded hard work while believing that success came with effort.

In our study the handball players who perceived a high-mastery motivational climate had more task-centered goal orientations, enjoyed practicing their sport more, and put more effort into training than handball players who perceived a high-performance motivational climate. These handball players obtained high scores in items suggesting an ego-centered goal orientation and a more apathetic approach to their sport including a willingness to use deception techniques.

With regard to the objective of our study, it seems that there is a relationship between the motivational climate created by the coach in the training sessions and the goal orientation, intrinsic satisfaction, and beliefs about the causes of success on handball players. This means that those players who perceived a mastery-orientated motivational climate showed a more positive goal achievement pattern than those who perceived a performance-orientated motivational climate. In the latter type of situation, players are punished for, rather than learning from, their errors and feel that for their coach the main thing to strive for is victory through improved performance and demonstrating their abilities.

Studies have shown the improved psychological well-being of players subjected to a mastery-oriented motivational climate compared to a more competitive training climate, which has been related to anxiety and reduced satisfaction with their sport (Balaguer et al., 1999, 2002; Pensgaard and Roberts, 2000; Duda, 2001; Vazou et al., 2006). In the study by Balaguer et al. (2002), it was observed that when handball players perceived an intensely task-oriented motivational climate, their performance and self-satisfaction with this performance improved, and their image of the coach was even better. In contrast, the perception of an ego-oriented motivational climate was negatively associated with ratings of their coach, yet in general, positively associated with satisfaction with their team’s results.

As in earlier studies (Seifriz et al., 1992; Walling et al., 1993; Escartí et al., 1999; Cervelló and Santos-Rosa, 2001; Vazou et al., 2005), our results reveal associated constructs of similar goal perspective.

In general, handball players who were self-satisfied with their sport were those subjected to the more mastery-orientated tasks while those whose training climate was more performance-orientated expressed their dissatisfaction and lack of enthusiasm or boredom. Other authors have also reported that the satisfaction of players with their sport’s progress and competition results is positively linked to a task-orientated motivational climate created by the coach (Treasure and Roberts, 1998; Balaguer et al., 1999, 2002; Ruiz-Juan et al., 2010; Gómez-López et al., 2014). In contrast, the negative feelings of players can lead to sport dropout, especially if there is a sense that small hurdles cannot be overcome and players question their own ability (Duda et al., 1995; Cervelló and Santos-Rosa, 2001; Castillo et al., 2002; Walling et al., 2002; Cecchini et al., 2004).

Lastly, for handball players who perceived a mastery-orientated training climate, defeat was viewed as part of the learning process and success, both in training and competition, was attributed to effort, while according to those perceiving a performance motivational climate, ability and deception techniques were the means to success. These findings are in agreement with those described by Seifriz et al. (1992) and Escartí et al. (1999).

## Conclusion

Specifically, the results of this study indicate that the coach’s socializing role determines a handball player’s behavior, sport commitment, and enjoyment. Although players show a personal predisposition for adopting a given goal orientation and behavior pattern (Cecchini et al., 2004). Our data confirm the relationship, both positive and negative, that coaches may have on their players. However, taking into account that they are young handball players, it is necessary to clarify that the training history (clubs, etc.) of these young players should be studied to better understand the variables evaluated in this work. Thus, the coach should create a task-orientated motivational climate (Gano-Overway et al., 2005), emphasizing self-reported achievements related to mastering skills, effort, and interest in the activity itself (Cecchini et al., 2004), while giving priority to displays of effort besides outcomes (Moreno et al., 2007). The coach should also encourage players to analyze their failures and successes as a way of self-satisfaction (Cervelló et al., 1999) and enhancing commitment rather than only of improving results.

As limitations to our study, we should mention that although our data extend the information provided in similar studies, the small sample size examined limits their generalization. These data require confirmation in future work. Further topics that could be addressed in the future are the influence on players of parents and peers, and the difference between an athlete’s and coach’s own perception of the coach-created motivational climate.

## Author Contributions

AG-G elaboration of method and results. MG-L elaboration of the introduction, discussion, and conclusions. NR-S reviewed the results. JA reviewed the introduction. MA reviewed the discussion and conclusions. AB studied design and reviewed the methods.

## Conflict of Interest Statement

The authors declare that the research was conducted in the absence of any commercial or financial relationships that could be construed as a potential conflict of interest.
